# BIS-11A -Hindi version: A preliminary study of impulsivity in rural and urban Indian adolescents

**DOI:** 10.4103/0019-5545.42395

**Published:** 2008

**Authors:** Paramjeet Singh, R. K. Solanki, P. S. Bhatnagar

**Affiliations:** Department of Psychiatry, SMS Medical College and Hospital, Jaipur, Rajasthan - 302 004, India

**Keywords:** Adolescent, BIS-11 A, Hindi, rural, urban

## Abstract

**Context:**

Despite of there being a pressing need to gauge impulsivity scores, there is no behavioral instrument in India to assess the impulsivity in adolescents. No earlier studies have been done in India to access impulsivity in adolescents. Even in western countries, no study has been done in rural setting to access impulsivity, although segment of rural population is small in western nations with major population residing in urban areas.

**Aims:**

To translate BIS-11A into Hindi from English in a culturally sensitive manner and to do preliminary study in rural and urban areas.

**Settings and Design:**

First translation of BIS-11 (as it is meant for adults) and cultural substitution resulted in Hindi adult version. Adolescent version was derived from adult version by replacing adult activities with adolescent activities.

**Materials and Methods:**

BIS-11 English version was translated into Hindi and a back translation was made. As BIS-11 was developed for adults, answering some of the questions poses challenges for adolescents, so to be used with adolescents, questions that do not fit into adolescent age group were substituted keeping in view the activities of adolescents. Besides, questions that were not suitable as per the Indian culture were modified. Initially, these changes were made hypothetically by discussion among the authors and later a group of 48 school students were interviewed about the questions. Based on the interviews of students a final version was prepared. Translation, back translation, cultural substitution -hypothetically, and in school by discussion were carried out. The questionnaire was given to 120 urban high school students (in Jaipur, northern India) and 50 rural students (at Kanota, 25 km from Jaipur, northern India) and the scores were calculated as per the scoring method provided with original BIS-11.

**Statistical Analysis:**

*T*-test (two-tailed, two sample unequal variance, i.e., type 3) was used.

**Results:**

*T*-test (two-tailed, two sample unequal variance, i.e., type 3) found no significant difference between impulsivity scores of adolescents of urban and rural areas *t* 0.05(2)1 = 0.57, |*t*| < *t* 0.05(2)1, *P* > 0.05, *P* = 12.706. There were no gender related differences either.

**Conclusions:**

As impulsivity can lead to suicide and is implicated for substance abuse in disorders like Schizophrenia, it is important that culturally sensitive impulsivity studies are done in India on a large scale keeping in view the large size of population. Standardization of the BIS11-A Hindi version is being taken up. The work on Hindi version also generates necessity for other tasks if BIS-11(Hindi version) is to be used widely. Work on psychometric properties of Hindi version of BIS-11 A is being taken up. There is a need to devise a quick way to calculate impulsivity scores keeping in view the large population of India (1 billion out of which at least 33% is Hindi speaking, Census Survey of India, 2001). Besides, BIS-11A needs to be developed for other regional languages in India as there is a high-linguistic diversity in India.

## INTRODUCTION

There is no behavioral instrument in India to gauge the impulsivity in adolescents and no earlier studies have been done in India to access impulsivity in adolescents. Even in western countries, no study has been done in rural setting to access impulsivity, although segment of rural population is small in western nations with major population residing in urban areas.

There are a number of instances in day-to-day life when happenings due to impulsivity come to notice such as persons committing suicide after examination results or jumping in front of a running train to die. Impulsivity and sensation seeking are involved in a wide spectrum of psychopathologic and social challenges which are a part of impulse control disorders. The significance of managing impulsivity can be gauged from the answers given by inmates who committed acts of aggression in prison despite of knowing that outcomes will not be desirable as they will be moved to less desirable living conditions and are less likely to be considered for parole. The inmates answered that we can't help it, we just do it.[1]

Barratt[2] has illustrated impulsivity with four different and basic categories of concepts that are used to describe people: biologic, cognitive, environmental, and behavioral. As impulsiveness has a biologic basis, BIS-11 was chosen as it is a questionnaire which has a biologic basis.[3] Besides it has also been revised extensively.[4] Earlier Italian,[5] Japanese,[6] and French[7] version have been developed on the basis of BIS-11 English version.

India is a vast country with all kinds of diversity, geographic, cultural, linguistic, and socioeconomic. Hindi is spoken by a majority of people particularly in the northern India and is the national language as per the Indian constitution. Although, one gets to see cases involving impulsivity in both clinical settings as well as in day-to-day life, e.g., newspapers and television, there is no behavioral instrument that can reliably measure impulsiveness among Indian people. India's vast population has a large adolescent segment (unlike many western countries where the population of older people is in majority); therefore, in the present study it was decided to derive a culturally sensitive version based on the BIS-11 for Indian adolescents. As India has 70% people living in rural areas, it was considered worthwhile to apply the same on rural adolescents besides urban as urban areas are fast growing due to migration of people from rural to urban areas so that a comparative view of impulsivity in rural and urban adolescents can be obtained.

## SUBJECTS AND METHODS

BIS-11 English version was translated into Hindi and a back translation was made. As BIS-11 was developed for adults, answering some of the questions poses challenges for adolescents,[5] so to be used with adolescents, questions that do not fit into adolescent age group were substituted keeping in view activities of adolescents. Besides, questions that were not suitable as per the Indian culture were modified. Initially, these changes were made hypothetically by discussion among the authors and later a group of 48 school students were interviewed about the questions. Based on the interviews of students, a final version was prepared. Translation, back translation, cultural substitution - hypothetically and in school by discussion were carried out. The questionnaire was given to 120 urban high school students (in Jaipur, northern India) and 50 rural students (at Kanota, 25 km from Jaipur, northern India) and the scores were calculated as per the scoring method provided with original BIS-11. Zar was consulted for statistical purpose.[8]

## RESULTS

BIS-11 was given to 125 urban adolescents and 50 rural adolescents. Their calculated values are shown in Table 1.

**Table 1:** Average impulsivity scores of boys and girls of rural areas along with standard deviation (SD)

		Cognitive	Nonplanning	Motor	Total
Rural girls	Mean	17.20	24.29	20.79	63.25
	SD	3.52	4.30	3.90	10.53
Rural boys	Mean	17.17	23.17	20.56	60.91
	SD	2.70	4.66	4.58	9.82

The values of cognitive, nonplanning, and motor scales along with total impulsivity scores have been shown in Tables 1-4. *T*-test (two-tailed, two sample unequal variance, i.e., type 3) found no significant difference between impulsivity scores of adolescents of urban and rural areas *t* 0.05(2)1 = 0.57, |*t*| < *t*0.05(2)1, *P* > 0.05, *P* = 12.706, thus with null hypothesis of no difference between the two samples was accepted.

**Table 2:** Average impulsivity scores of boys and girls of urban areas along with standard deviation (SD)

		Cognitive	Nonplanning	Motor	Total
Urban girls	Mean	16.06	23.04	21.40	60.48
	SD	3.04	4.11	3.82	8.84
Urban boys	Mean	17.26	23.67	22.97	64.18
	SD	2.64	4.48	3.76	8.61

**Table 3:** Averages in impulsivity scores of adolescents of rural and urban areas

	Cognitive	Nonplanning	Motor	Total
Rural	17.21	23.71	20.89	61.71
Urban	16.82	23.41	22.32	62.65

**Table 4:** Standard deviation in impulsivity scores of adolescents of rural and urban areas

	Cognitive	Nonplanning	Motor	Total
Rural	3.14	4.51	4.25	9.46
Urban	3.72	19.79	12.91	9.12

For gender differences, *t*-test scores between rural boys and girls was insignificant *t* 0.05(2), 1 = 0.94, |*t*| < *t*0.05(2)1, *P* > 0.05, *P* = 12.706), resulting in acceptance of null hypothesis. Likewise, between urban boys and girls no significant difference was found. *t* 0.05(2),1 = 0.02, |*t*| < *t*0.05(2)1, *P* > 0.05, *P* = 12.706.

## DISCUSSION

Impulsiveness and irritability (anger) are both potential criteria for an antisocial personality disorder.[9] Impulsiveness and verbal skills are inversely related[10] from society for biologic psychiatry - 1997 and positively related to poor judgments of time duration[10] and is also related to morningness.[11]

Impulsivity is involved in a number of important and widely spread psychiatric disorders. It is one of the main dimensions of suicidality and all the three dimensions of impulsivity (behavioral loss of control, nonplanning, and cognitive) are involved in severely depressed patients.[12] High levels of impulsivity and sensation seeking are also associated with substance in schizophrenia patients.[13] Impulsivity is also prominent characteristic of bipolar disorder and severe suicidal behavior is associated with increased impulsivity.[14] Both impulsivity and emotional distress are related to risk taking in gamblers. Besides younger age and impulsivity are known as risk factors for illegal activities.[15]

In the present study , *t*-test did not show any significant difference between rural and urban adolescents, therefore, the present translation can be used both with rural and urban adolescents. One of the important factors for this could be that the influence of literacy and TV is growing in rural areas. Due to this, in urban as well as rural areas, gender differences are reducing, girls are acquiring education at par with boys and also their role in other spheres of day-to-day life is increasing in socioeconomic spectrum, these accounts for no significant gender difference in impulsivity scores in urban boys and girls.

In developing Hindi adolescent version, 11 questions needed change. These reflected differences in Indian cultural and socioeconomic lifestyle as compared to US and Italian scenario. In comparison to Italian version 7 questions (Nos. 10, 11, 13, 20, 24, 25, and 29) were different, 7 and 16 differed slightly (i.e., were similar) and 26 and 28 were same [Table 5]. Fossati *et al.*[5] required to reword 15 of the 30 BIS–11 items, with minor modifications being made to 11 of the 15 problematic items. Similarity, between Hindi and Italian versions is arising out of the fact that both the versions were for adolescents and were being derived from adult version of translated BIS–11. Dissimilarities between Italian and Hindi version were due to different socioeconomic and cultural settings in India as compared to Italy. Like Italian version, BIS–11 (Hindi) version also maintains a 30-item, Likert type self-report format. All items were measured on a four-point ordinal scale (1 = Rarely/never, 2 = occasionally, 3 = often, 4 = almost always/always) Four usually shows most impulsive response. The items were summed and impulsiveness was directly proportional to impulsivity score. Back translation was made to ensure that the questionnaire is adequate with respect to original versions, i.e., same as in Italian version.[5]

**Table 5:** Comparison of BIS-11 with Italian and Hindi versions

S. no.	Barratt scale BIS-11	Italian	Hindi-adult*	Hindi adolescent*
1	I plan tasks carefully	I plan what I have to do	Same	Same
7	I plan trips well ahead of time	I plan my spare time	Same	I plan to spend my spare time
10	I save regularly	Same as BIS-11	Same	I spend money in a planned way
11	I “squirm” at plays or lectures	Same as BIS-11	Same	I “squirm” in cinema or school/educational, institution
13	I plan for job security	Same as BIS-11	While having one source of livelihood, I collect information about other means of livelihood as well	I plan for my future or job or means of livelihood
16	I change jobs	I change my mind about what I will do when I grow up	I change means of livelihood	I change plans as to what career will I adopt when I grow up
20	I am a steady thinker	Same as BIS-11	I am a steady thinker	I am a person who dwelves a lot in thinking
21	I change where I live	I change friends	Same	I keep on changing my friends
24	I change hobbies	Same	Same	I change my hobbies, sports, part-time job/part-time profession
25	I spend or charge more than I earn	I spend more than I should	I spend more than I earn	I spend without planning
26	I often have extraneous thoughts when thinking	When I think about something, other thoughts pop up in my mind	When I think about something, other thoughts pop up in my mind	When I think about something, other thoughts pop up in my mind
28	I am restless in lectures or talks	I am restless at movies or lectures	I am restless in talks or discussions	I am restless in cinema or lecture
29	I like puzzles	Same	I like solving difficult matters	I like solving cross-words puzzle or playing chess

* Means change of gender in the Hindi version

Male adolescents showed significantly higher mean BIS–11 score than that of female adolescents in Italian version,[5] which is unlike the Hindi version in the present study. This may be due to larger sample size in case of Italian study (563 adolescents; out of which 209, i.e., 37.1% male while 354 subjects, i.e., 62.9% female), as in case of urban adolescents (Total 120 adolescents; *n* = 72 for boys, i.e., 60% and 48, i.e., 40% for girls) and for rural subjects (50 adolescents, 25 boys, and 25 girls; 50% each).
